# Diagnosis, differential diagnosis, and treatment for sudden sensorineural hearing loss: Current otolaryngology practices in China

**DOI:** 10.3389/fneur.2023.1121324

**Published:** 2023-02-23

**Authors:** Nishan Chen, Niki Karpeta, Xin Ma, Xianhui Ning, Xiaoling Liu, Jijun Song, Zigang Jiang, Xiulan Ma, Xiuli Liu, Shixun Zhong, Qing Sun, Jun Liu, Ganggang Chen, Maoli Duan, Lisheng Yu

**Affiliations:** ^1^Department of Otolaryngology, Head, and Neck Surgery, Peking University People's Hospital, Beijing, China; ^2^Department of Otolaryngology, Head, and Neck Surgery, Karolinska University, Solna, Sweden; ^3^Department of Otolaryngology, Head, and Neck Surgery, Zhongshan Hospital, Shanghai, China; ^4^Department of Otolaryngology, Inner Mongolia People's Hospital, Hohhot, Inner Mongolia, China; ^5^Department of Otolaryngology, Zhoukou Central Hospital, Zhoukou, China; ^6^Department of Otolaryngology, First Hospital of Qinhuangdao, Qinhuangdao, China; ^7^Department of Otolaryngology, Head, and Neck Surgery, Sheng Jing Hospital, Shenyang, China; ^8^Department of Otolaryngology, First Affiliated Hospital of Dalian Medical University, Dalian, China; ^9^Department of Otolaryngology, First Affiliated Hospital of Chongqing Medical University, Chongqing, China; ^10^Department of Otolaryngology, Head, and Neck Surgery, Third Medical Center of PLA General Hospital, Beijing, China; ^11^Department of Otolaryngology, Head, and Neck Surgery, Chinese PLA General Hospital, Beijing, China; ^12^Department of Otolaryngology, Head, and Neck Surgery, First Hospital of Shanxi Medical University, Taiyuan, China; ^13^Department of Clinical Science, Intervention and Technology, Karolinska Institute, Stockholm, Sweden

**Keywords:** sudden sensorineural hearing loss (SSNHL), otolaryngologists, corticosteroids (CS), survey questionnaire, hospital grade, differential diagnosis

## Abstract

**Introduction:**

Although sudden sensorineural hearing loss (SSNHL) has been attempted to be understood for 70 years, diagnosis and treatment strategies still have strong heterogeneity worldwide, which are reflected in the guidelines issued by countries and the clinical practice of otolaryngologists.

**Methods:**

Questionnaires were sent to registered otolaryngologists nationwide *via* an online questionnaire system. We investigated the current views and clinical practices of otolaryngologists in mainland China about the diagnosis, examination, and treatment strategies of SSNHL.

**Results:**

Most otolaryngologists supported diagnostic classification *via* audiograms. Regional economic situation and hospital grade affected application strategies for differential diagnosis. Regarding corticosteroid therapy, 54.9% of respondents opted to discontinue the drug 5 days after systemic administration. Both intratympanic therapy and post-auricular injections were selected by more than half of the respondents as initial and salvage treatments.

**Discussion:**

Chinese otolaryngologists exhibit heterogeneity in clinical practices for SSNHL, including distinct approaches to combination therapy and local application of steroids. This study pointed out Chinese doctors' similarities, differences, and unique strategies in diagnosing and treating SSNHL and analyzed the possible reasons to help the world understand the current otolaryngology practices in China.

## 1. Introduction

The concept of sudden sensorineural hearing loss (SSNHL) was first proposed in 1944 (1). SSNHL is defined as an unexplained sensorineural hearing loss occurring within 72 h (2). Numerous clinical and basic studies have examined the etiology, diagnostic criteria, diagnostic tests, and treatments such as corticosteroids for SSNHL. Further, several countries have updated the guidelines for SSNHL in recent years (3–9). Nevertheless, there are significant differences in diagnostic and treatment strategies due to the low quality of available evidence.

We focused on guidelines published by China, the US, Japan, the UK, Germany, Spain, and other countries and found large differences in diagnostic criteria (Table 1). In addition, some countries recommend the clinical classification of SSNHL based on the pure-tone test (2, 4), while others do not (6, 8). Regarding supplementary examination, the recommendations of guidelines are also different. Although glucocorticoids are recognized as an effective treatment of SSNHL, the recommended route of delivery, dosage, and period of administration still have heterogeneity. Moreover, the effectiveness of hemorheological drugs remains controversial. As a result of these differences, clinicians may hold personal views and follow different instructions in their clinical practice, which is not conducive to standardized treatment and high-quality RCT research. For example, the inclusion and exclusion criteria may be inconsistent, caused by the differences in diagnostic criteria and clinical classification. As a result, the conclusions of similar RCT studies deviate considerably. Similarly, different dosages and treatment periods make it difficult to include similar research in meta-analyses.

**Table 1:** Comparison of key information in SSNHL guidelines.

**Nation**	**China**	**United States**	**Germany**
Agency	CMA	AAO-HNS	AWMF
Year of publication	2015	2019	2014
Audiological criteria	A decrease ≥20 dB affecting at least 2 consecutive frequencies	A decrease ≥30 dB affecting at least 3 consecutive frequencies	Not specified
Classification	Classified into four types by frequency and severity of hearing loss	Not specified	Classified into five types by frequency and severity of hearing loss
Supporting test	*Necessary*: Otoscopy, Tuning fork test/Pure tone test, Nystagmus examination (when accompanied by vertigo) *As required*: OAE/ABR /ECochG, Imaging examination, Laboratory testing.	*Recommendation*: History and physical examination, Audiometry, Retrocochlear pathology (MRI/ABR) *Strong recommendation against:* CT of the head, Laboratory tests	*Necessary*: History, Otoscopy, Tuning fork test/Pure tone test, Nystagmus examination *As required*: OAE/Stapedius reflex, Cervical spine, Imaging examination, Laboratory testing. ASSR, etc.
Steroid therapy	*Systemic application:* prednisone at 1 mg/kg/d for 3-5 days *Local application*: ITS/PAS as salvage treatment	*Systemic application*: prednisone at 1 mg/kg/d for 7-14 days, then taper over *Local application*: ITS as initial or salvage treatment	*Systemic application*: prednisolone at 250mg/d for 3 days *Local application*: ITS as salvage treatment
Combination therapy	*Recommendation*: Vasodilators and Hemorheology *Against*: Hyperbaric oxygen therapy	*Optional*: Hyperbaric oxygen therapy *Strong recommendation against*: Other pharmacologic therapy	*Optional*: Vasodilators and Hemorheology *Not specified*: Hyperbaric oxygen therapy *Against*: Hydroxyethyl starch

CMA, Chinese Medical Association; AAO-HNS, American Academy of Otolaryngology-Head and Neck Surgery; AWMF, Arbeitsgemeinschaft Wissenschaftlich Medizinischer Fachgesellschaften (Association of Scientific Medical Societies in Germany); OAE, Otoacoustic emission; ECochG, electrocochleogram.

As China is large and the most populated country, applying the guidelines to different levels of hospitals in 31 provincial bureaus is difficult. The guidelines only provide basic diagnosis and treatment proposals in most cases; local hospitals and otolaryngologists should also make necessary adjustments according to their conditions and the personalized needs of patients. Thus, it is necessary to understand individual preferences and local practices of otolaryngologists in China nationwide. These types of surveys in the UK, US, Germany, and Austria have been published (10, 11). Regarding these questionnaires, we modified a survey to Chinese-specific situations. Our survey focused on the current preferences and opinions of Chinese otolaryngologists regarding the diagnosis and treatment of SSNHL. According to the Ministry of Health of China, hospitals in China are classified into primary (Level 1), secondary (Level 2), and tertiary (Level 3) hospitals according to their standards of medical care, education, and research. Further, secondary (Level 2) and tertiary (Level 3) hospitals are classified into Grades A, B, and C based on their size, technology, equipment, and management. Otolaryngologists in different hospitals may employ divergent medical strategies. We explored potential factors influencing their clinical decisions and compared our results with those of other countries (10, 11).

At present, the uniqueness of the SSNHL practice in China has not been well-known worldwide. We believe that our findings revealed the current practice of Chinese physicians and provided new information, including the proposal and application of post-aural steroid delivery and the combination therapy of hemorheology drugs. We hope this information could arouse the interest of peers worldwide and provide future research ideas for RCT research design.

## 2. Materials and methods

Our questionnaire (Supplementary material 1) was originally based on the design of Sutton et al. (10) and Lechner et al. (11), and was modified according to Chinese-specific situations and the uniqueness of clinical practice. An online questionnaire system sent a survey link to otolaryngologists in mainland China. The survey was performed in accordance with relevant regulations in China and was approved by the Ethics Committee of Peking University People's Hospital (2019PHB109-01). All participants received an informed consent form on the front page of the questionnaire, and participants could not fill in the questionnaire until they signed it. The inclusion criteria were otorhinolaryngologists who hold a practicing certificate issued by the government health department. The exclusion criteria were doctors who could not use intelligent communication devices, doctors who were not qualified for various reasons, and doctors who had not done actual medical work within 1 year.

China has vast national land and uneven population distribution with complex governmental divisions and developing informational networks. Currently, the government's complete contact lists of otolaryngologists in China are unavailable. Thus, we sent our survey link to every provincial organization of physicians and received responses from all interviewees. Although the exact number of doctors who received the questionnaire was difficult to quantify, our survey was considered to approximate nationwide research.

In total, 2015 respondents participated in the survey and completed all questionnaires. The survey was divided into several parts, encompassing basic information, diagnostic criteria, systemic and local steroid therapy, and combination therapy. The interviewed otolaryngologists were required to respond in view of their clinical practice.

All topics were presented as single- or multi-choice questions. In cases where the respondent perceived no optimal option, they were allowed to select “other” and fill in the content autonomously. The responses were submitted and included in statistical analysis upon completing the questionnaire.

Categorical variables were expressed as count and percentage. Univariable ordinal logistic regression models and a multivariable ordinal logistic regression model were performed to evaluate factors influencing classification diagnosis. All these statistical analyses were performed using SPSS 23.0 software (SPSS Inc, Chicago, IL, USA). All *P*-values were 2-tailed, and *P* < 0.05 was considered statistically significant. The power of statistical analysis was calculated using PASS 23.0 software. Graphs were generated using GraphPad Prism 7.

## 3. Results

### 3.1. Questionnaire recovery and distribution

China is the most populated country, and its mainland is divided into 31 provincial bureaus. The sample sizes of each province are presented in Table 2 and mapped in Supplementary material 2. The total number of licensed otolaryngologists in mainland China is ~30,000. The sample size of this study was considered sufficient, given that the effective number of questionnaires collected in this study was 2015, which achieves 100% power to detect logistic regression models by a posteriori validation. All required questions were answered in each questionnaire, but some questions were skipped automatically by the predesigned logic system. Thus, the number of valid answers varied among questions.

**Table 2:** Number of questionnaires in different provinces of China.

**Province**	**Number**	**Province**	**Number**
Beijing	107	Hainan	8
Shanghai	69	Henan	120
Tianjin	15	Xinjiang	71
Jiangsu	66	Sichuan	208
Zhejiang	102	Hebei	179
Fujian	89	Qinghai	14
Guangdong	98	Anhui	47
Shandong	72	Jiangxi	17
Inner Mongolia	72	Shanxi	117
Hubei	31	Heilongjiang	61
Chongqing	72	Tibet	8
Shaanxi	33	Guangxi	36
Liaoning	131	Guizhou	13
Jilin	29	Yunnan	39
Ningxia	58	Gansu	10
Hunan	23	**Total**	**2015**

**Figure 1:**
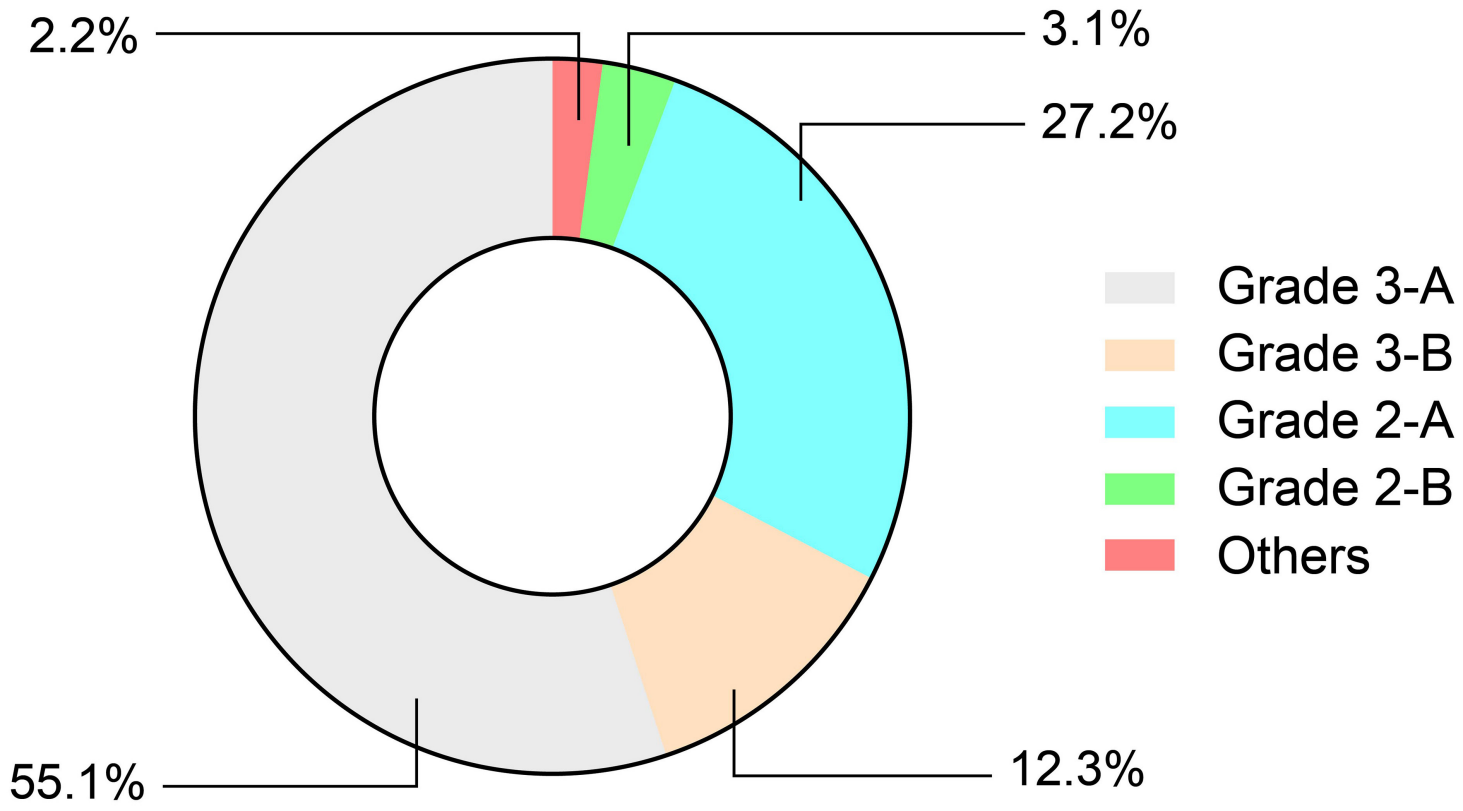
Distribution of hospital grade.

Most of the 2015 respondents were from 3-A hospitals (55.1%, *n* = 1,110) and 2-A hospitals (27.2%, *n* = 549). Other respondents were from 3-B hospitals, 2-B hospitals, and other types of hospitals (Figure 1). Otolaryngologists reported different clinical experiences and professional titles in China. Of respondents, 13.6% (*n* = 275) were within 5 years of employment, 17.4% (*n* = 350) were within 5–10 years, 34.1% (*n* = 688) were within 10–20 years, 31.9% (*n* = 642) were within 20–35 years, and 3.0% (*n* = 60) had been employed for more than 35 years. In terms of professional titles, the percentages of residents, specialists, senior consultants, and consultants were 14.6% (*n* = 295), 32.1% (*n* = 646), 32.7% (*n* = 660), and 19.1% (*n* = 384), respectively.

**Figure 2:**
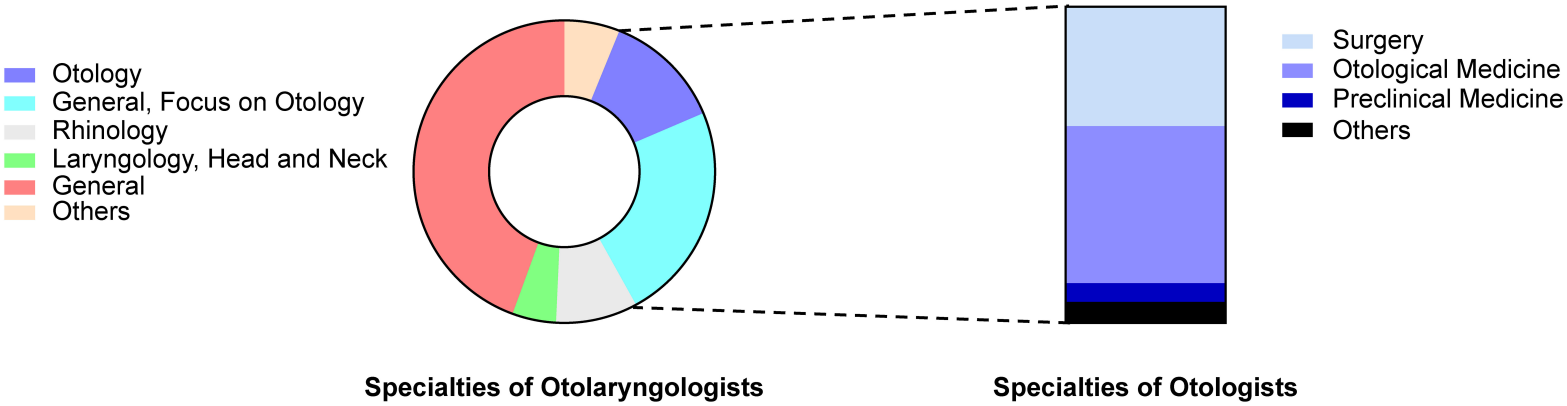
Distribution of research fields of respondents.

Regarding specific research fields, most respondents were general otolaryngologists (68.7%; *n* = 1384) without specific subspecialties. Approximately one-third (*n* = 469) of these doctors indicated that their clinical work was predominantly in otology. Of the respondents, 25.7% (*n* = 517) declared a sub-disciplinary research field; 256 were otologists, and the others worked in the nasal, throat, or neck surgery field. Among otologists, 37.8% were in surgery, 49.6% were in otology and audiology, 6.1% were in basic medical sciences, and 6.5% were in other fields, such as hearing detection, neuroscience, and Chinese medicine (Figure 2).

As a developing country, the economic situation in China varies among regions, which is a key factor causing unbalanced medical resources across the country. To assess the regional representativeness of the survey, we calculated the population composition ratio in different economic regions and compared it with our sample composition ratio using a Chi-square test (Table 3). The sample distribution in this study was slightly lower than expected in developed regions, was higher than expected in moderately developed regions, and was in line with the expected value in less developed regions.

**Table 3:** Demographic distribution of surveys.

**Economic pattern**	**Population (10, 000)**	**Observed N./Proportion**	**Expected N./proportion**	**Chi-square**
Developed	53,711	721/35.8%	860.0/41.4%	50.57
Moderately developed	42,983	753/37.4%	621.1/29.9%	
Less developed	40,322	541/26.8%	533.9/25.7%	

### 3.2. Diagnostic criteria and classification

Most doctors (93.1%, *n* = 1,875) had sufficient clinical experience in SSNHL and handled at least 2–3 cases of SSNHL per month. More than half of the doctors responded that the most commonly encountered consultations were for patients with sudden hearing loss occurring within 14 days and without prior treatment (58.1%, *n* = 1171). In contrast, one-third of the doctors responded that consultation time varied substantially (32.5%, *n* = 655).

**Figure 3:**
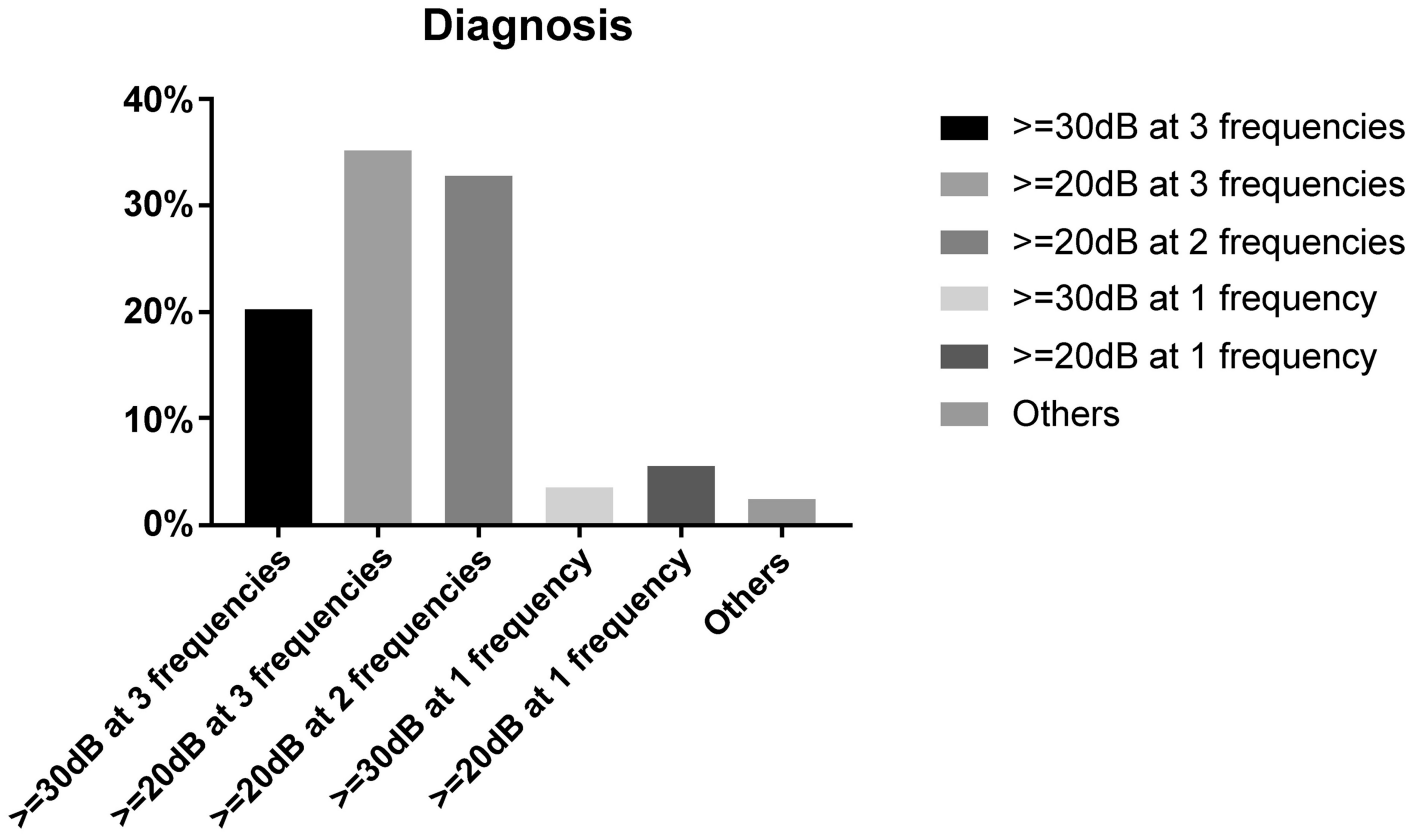
Criteria used to define sudden sensorineural hearing loss (SSNHL).

Chinese doctors reported different opinions regarding SSNHL diagnosis. Of respondents, 32.7% (*n* = 659) defined SSNHL based on the latest Chinese guidelines, i.e., ≥ 20 dB of hearing loss in two consecutive frequencies. Of doctors, 20.3% (*n* = 409) supported the criteria of AAO-HNS, i.e., hearing loss ≥ 30 dB in three continuous frequencies (Table 1). The definition of cases with “hearing loss ≥ 20 dB in at least three frequencies” received the most recognition (35.3%, *n* = 712); this definition falls between the Chinese and American criteria but is not mentioned in any guidelines. Less than one-tenth of doctors defined SSNHL as cases with hearing loss of 30 dB or 20 dB at any frequency. Other rare opinions included a 15 dB or 40 dB hearing loss or based on patient complaints only (Figure 3).

Respondents generally agreed on the key role of classification in SSNHL diagnosis and treatment. In a multi-choice questionnaire, 72.9% (*n* = 1,470) of respondents indicated that clinical classification helped them to explain the possible pathogenesis to patients, 76.7% (*n* = 1,546) indicated that classification helped estimate patient outcomes, and 72.7% (*n* = 1,465) indicated that they would choose different treatment strategies based on different classifications. Only 6.2% (*n* = 124) of respondents indicated that classification had no significant effect on clinical practice. However, classification diagnosis remains to be implemented in actual clinical practice. Our survey revealed that only 37.2% (*n* = 749) of physicians performed the classification procedure for every patient, and 33.8% (*n* = 680) were able to classify the most handled cases. Of physicians, only 13.5% (*n* = 273) performed classification occasionally, and 15.5% (*n* = 313) respondents never classified any SSNHL cases.

The division of economic regions was based on the Gross Domestic Product (GDP) rankings and populations of provinces issued by the National Bureau of Statistics in 2018. We calculated the per capita GDP of each province: regions ranked in the top 1–10 were classified as economically developed, regions ranked 11–20 were classified as moderately developed regions, and regions classified in the bottom 21–31 were classified as less developed regions (Table 4). We conducted a logistic regression analysis using SPSS 23.0 (Table 5) to investigate further the factors influencing classification diagnosis. In multivariate regression analysis, hospital grade, economic region, working experiences of doctors, and professional titles were considered. Hospital level was the only independent factor influencing clinical classification. Compared to top-level hospitals, respondents from lower-level hospitals were more likely to perform less or no classification in their clinical practice (OR = 0.452, 95% CI = 0.283–0.722, *p* = 0.001). The economic region, working experience, and professional title did not affect the inclination for classification.

**Table 4:** Regional economic patterns of China.

**Economic pattern**	**Regions**
Developed	Beijing, Shanghai, Tianjin, Jiangsu, Zhejiang, Fujian, Guangdong, Shandong, Inner Mongolia, Hubei
Moderately developed	Chongqing, Shaanxi, Liaoning, Jilin, Ningxia, Hunan, Hainan, Henan, Xinjiang, Sichuan
Less developed	Hebei, Qinghai, Anhui, Jiangxi, Shanxi, Heilongjiang, Tibet, Guangxi, Guizhou, Yunnan, Gansu

**Table 5:** Multi-factor analysis of diagnosis type.

**Factor**		**OR**	**95% CI**		***P***
			**Lower**	**Upper**	
Hospital grade	1	0.452	0.283	0.722	0.001^*^
	2	0.677	0.409	1.123	0.131
	3	0.884	0.549	1.423	0.611
	4	/	/	/	/
Region	1	0.997	0.806	1.232	0.975
	2	0.950	0.771	1.171	0.630
	3	/	/	/	/
Working experience	1	0.589	0.309	1.124	0.109
	2	0.793	0.516	1.217	0.288
	3	0.909	0.615	1.342	0.630
	4	0.823	0.573	1.184	0.294
	5	/	/	/	/
Professional titles	1	1.041	0.667	1.623	0.860
	2	1.004	0.678	1.488	0.983
	3	1.327	0.943	1.866	0.104
	4	/	/	/	/

* *p* < 0.05.

### 3.3. Supporting tests

**Figure 4:**
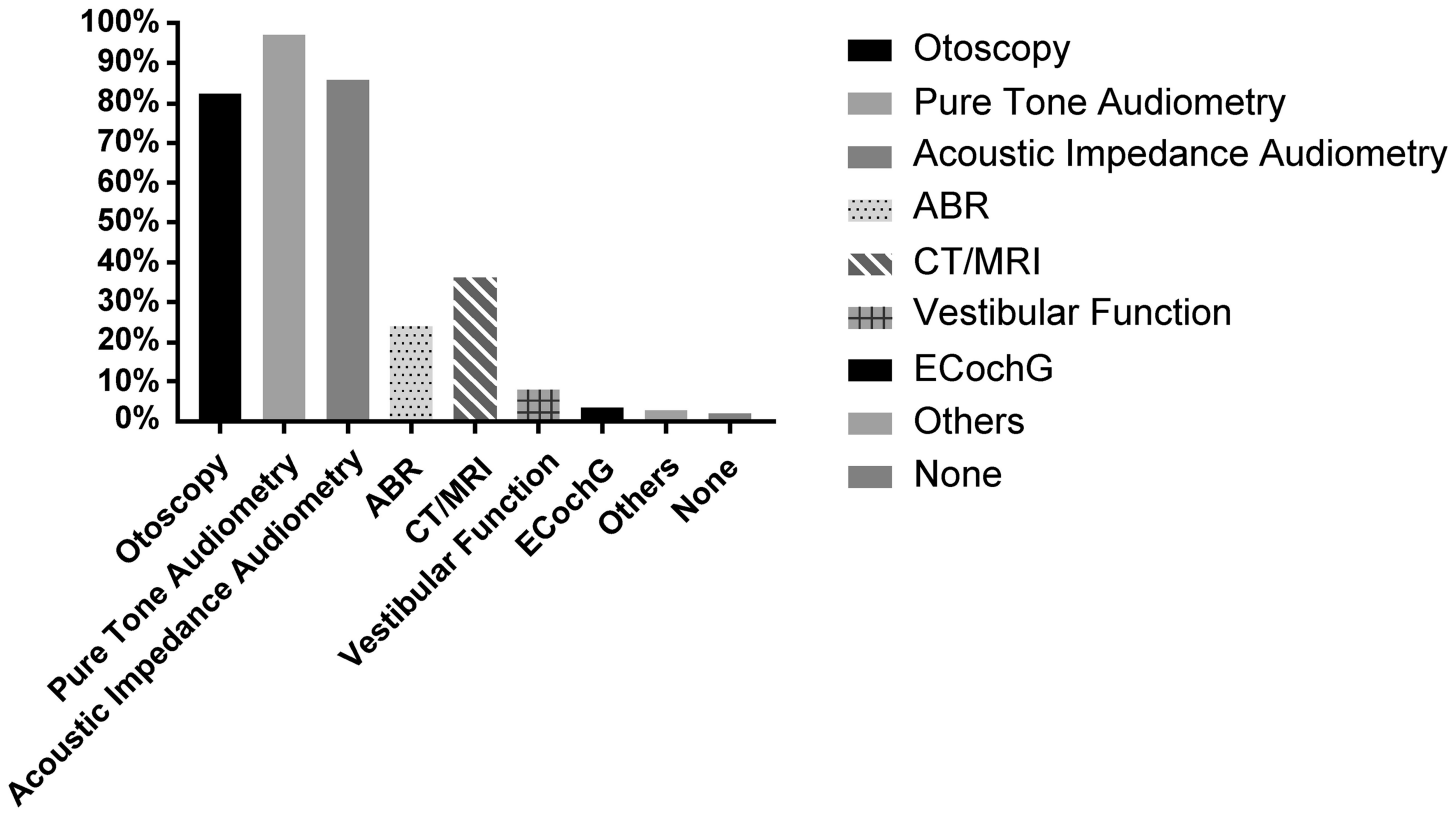
Support tests for differential diagnosis.

More than 80% of physicians always performed otoscopy, pure tone test, and acoustic impedance examinations; only 36.3% (*n* = 732) used imaging as part of routine examinations. A small proportion of physicians used ABR (23.9%, *n* = 481), electrocochleography (3.4%, *n* = 67), and vestibular function (7.8%, *n* = 157) in routine examinations. Only a few physicians (2.0%, *n* = 39) based their diagnoses on patients' complaints without examining them. In addition, several respondents indicated that otoacoustic emissions, extended high-frequency audiometry, and Eustachian tube function tests were included in their routine examinations (Figure 4).

Binary logistic regression analysis revealed that hospital level and economic situation were independent factors that affected whether doctors routinely performed ABR tests and cochlear electrogram examinations, respectively. Doctors in 3-A hospitals were more inclined to perform ABR tests than other hospitals (OR = 3.488, 95% CI = 1.365–8.915, *p* = 0.009). Doctors in less developed regions performed fewer audiology tests than those in more developed regions (OR = 0.517, 95% CI = 0.285–0.938, *p* = 0.030). Professional title and working experience did not significantly affect clinical decisions. For imaging examinations, none of these factors influenced whether MRI was performed. For the choice of ‘no tests,' only the professional title was an independent factor. Compared to consultants, lower-level doctors were more inclined to make diagnoses based solely on patients' complaints without performing any tests, which goes strongly against recommended guidelines (OR = 0.141, 95% CI = 0.025–0.800, *p* = 0.027) (Table 6).

**Table 6:** Binary logistic regression analysis of differential diagnosis.

**Test**	**Factor**		**OR**	**95% CI**		***P***
				**Lower**	**Upper**	
ABR	Hospital grade	1	3.488	1.365	8.915	0.009^*^
		2	2.020	0.758	5.383	0.160
		3	1.466	0.562	3.820	0.434
		4	/	/	/	/
ECochG	Region	1	0.517	0.285	0.938	0.030^*^
		2	0.545	0.305	0.974	0.040^*^
		3	/	/	/	/
No tests	Professional titles	1	0.141	0.025	0.800	0.027^*^
		2	0.134	0.026	0.686	0.016^*^
		3	0.131	0.026	0.669	0.015^*^
		4	/	/	/	/

* *p* < 0.05.

It is worth mentioning that the medical system in China is significantly different from those in Europe and the United States. For example, most patients in China directly turn to the otorhinolaryngology department of general hospitals, while European and American patients tend to make the first consultation in the community. Thus, there were certain differences between support tests in the first screening, which may affect doctors' judgment and treatment.

### 3.4. Treatments

#### 3.4.1. Steroid therapies

Our survey investigated the current applications of steroid therapies by Chinese otolaryngologists. When using systemic steroids, the most popular protocol was discontinuing the treatment after 5 days of intravenous application (40.5%, *n* = 816). Of respondents, 18.9% (*n* = 380) typically administered steroids orally and discontinued the medication after 5 days. The Chinese guidelines recommend both protocols. Some respondents selected intravenous application for 5 days and gradually reduced the dosage (24.8%, *n* = 499), whereas others preferred intravenous-oral sequential administration (10.4%, *n* = 210) and oral administration (5.5%, *n* = 110), followed by a gradual dosage reduction. With regard to the variety of steroids, methylprednisolone (54.4%, *n* = 1096) and dexamethasone (65.9%, *n* = 1328) were the most commonly used types of intravenous steroids. In comparison, prednisone (77.4%, *n* = 1560) and methylprednisolone (26.1%, *n* = 527) were the most commonly used types of oral steroids. Since respondents were allowed to select more than one type of drug, the total percentage exceeded 100%.

**Figure 5:**
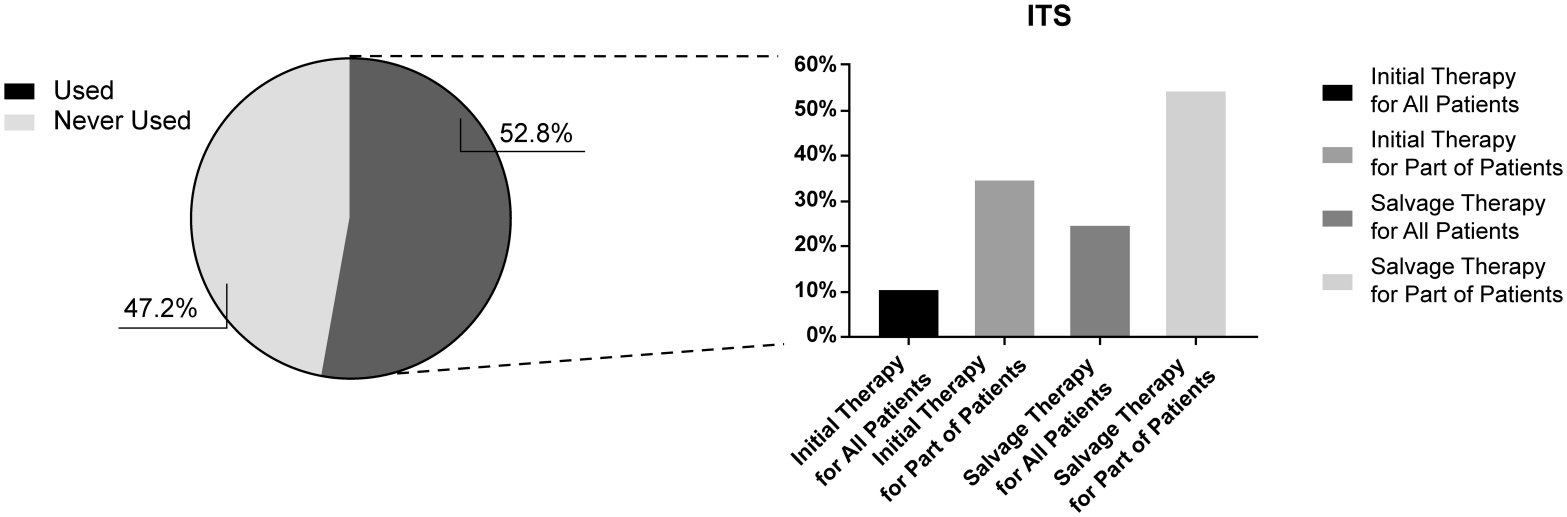
Application of intratympanic steroid injections.

Our survey also investigated the current applications of local steroid administration. The results indicated that 52.8% (*n* = 1664) of Chinese otolaryngologists had attempted ITS therapy, predominantly used as a salvage treatment. Specifically, 54.2% (*n* = 577) applied the treatment in patients who failed initial treatment; 24.5% (*n* = 261) preferred to attempt salvage treatment in all patients who failed initial treatment. Of the respondents, 34.8% (*n* = 370) used ITS as an initial treatment in specific patients, while 10.7% (*n* = 114) of respondents used it as an initial treatment for all patients (Figure 5).

**Figure 6:**
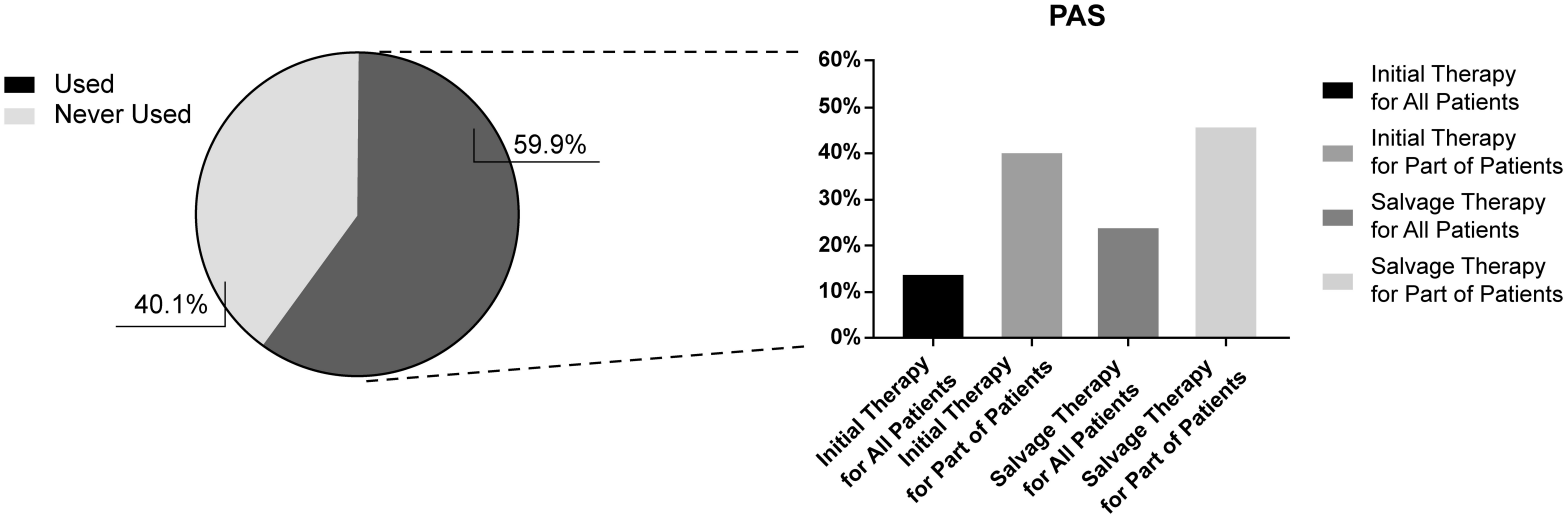
Application of post-aural steroid injections.

Besides ITS, another local drug delivery approach widely used in China was post-aural steroids (PAS) therapy. The procedure is operated by an injector that enters vertically from the skin at the mid-point of the retroauricular groove, delivering drugs into the periosteum of the mastoid process (12). Our survey is the first to investigate the use of PAS therapy by Chinese otolaryngologists. Of the respondents, 59.1% (*n* = 1919) had attempted post-auricular injections in their clinical practice, which exceeded the number of respondents who had used ITS, highlighting the convenience and popularity of post-auricular injections. Similarly, most doctors used PAS as a salvage treatment; 45.7% (*n* = 544) used it in a proportion of patients who failed initial treatment, and 23.7% (*n* = 282) attempted it in all patients who failed initial treatment. Of the respondents, 40.0% (*n* = 477) used it as an initial treatment in specific patients, and 13.8% (*n* = 165) used it as an initial treatment in all patients (Figure 6).

#### 3.4.2. Hemorheology treatment

Despite the vital role of steroid treatment for SSNHL, most Chinese doctors tended to use vasoactive and rheologic agents as combination treatments. Of the respondents, 61.7% (*n* = 1,243) reported using at least one type of combination medicine. In the investigation of SSNHL pathogenesis, cochlear ischemia, embolism, and vasospasm were approved by almost all physicians (97.1%, *n* = 1,957). Therefore, hemorheology treatment was considered a key treatment for SSNHL in China. Our survey investigated hemorheology drugs commonly used by Chinese otolaryngologists, which are listed in Table 7. Results of the multi-choice questionnaire revealed that extracts of *Ginkgo biloba* leaves (EGB) (91.2%, *n* = 1,134), alprostadil (55.6%, *n* = 691), and batroxobin (51.5%, *n* = 640) were favored by more than half of Chinese physicians. Thrombus (26.2%, *n* = 326), vinpocetine (22.5%, *n* = 280), and ShuXueTong (a type of herbal medicine) (6.4%, *n* = 80) were preferred by a proportion of physicians.

**Table 7:** Hemorheology drugs used by Chinese otolaryngologists.

**Drugs**	**Description**
GB 761^®^	Extract of Ginkgo biloba Leaves tablets used for microcirculatory disturbances
Alprostadil	Prostaglandin E1, which has vasodilatory properties.
Batroxobin	A snake venom enzyme used as a defibrinogenating agent.
Vinpocetine	A synthetic derivative of the vinca alkaloid vincamine. Mechanisms include blockage of sodium channels and antioxidant activity.
Shu Xuetong	A traditional Chinese drug used to ameliorate stagnation of blood flow.
Panax Notoginsenosidum	A traditional Chinese drug extract from *P. notoginseng* used for microcirculatory disturbances

**Figure 7:**
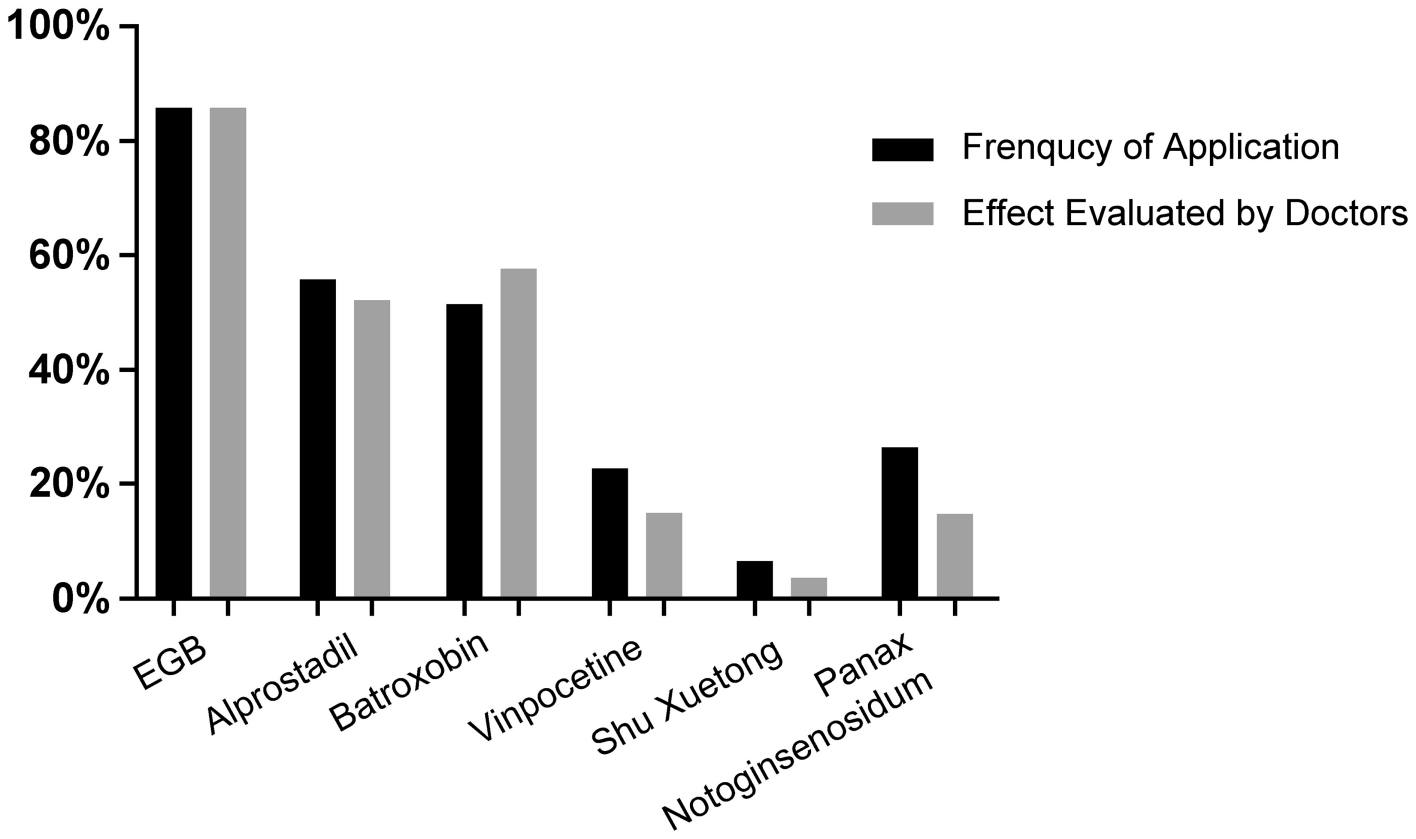
Application of hemorheology drugs.

In terms of efficacy evaluation, results were similar to those reported above, i.e., from high-efficacy to low-efficacy: EGB (85.8%, *n* = 1,066), batroxobin (57.8%, *n* = 718), alprostadil (52.1%, *n* = 648), thrombus (14.9%, *n* = 185), vinpocetine (14.9%, *n* = 185) and Shu Xuetong (3.7%, *n* = 46) (Figure 7). In particular, although batroxobin ranked third in usage frequency, it ranked higher in the efficacy evaluation. This could be due to the lack of reimbursement in some regions, cost, the complexity of the clinical application, and follow-up difficulties.

#### 3.4.3. Nutritional neurological drugs

**Figure 8:**
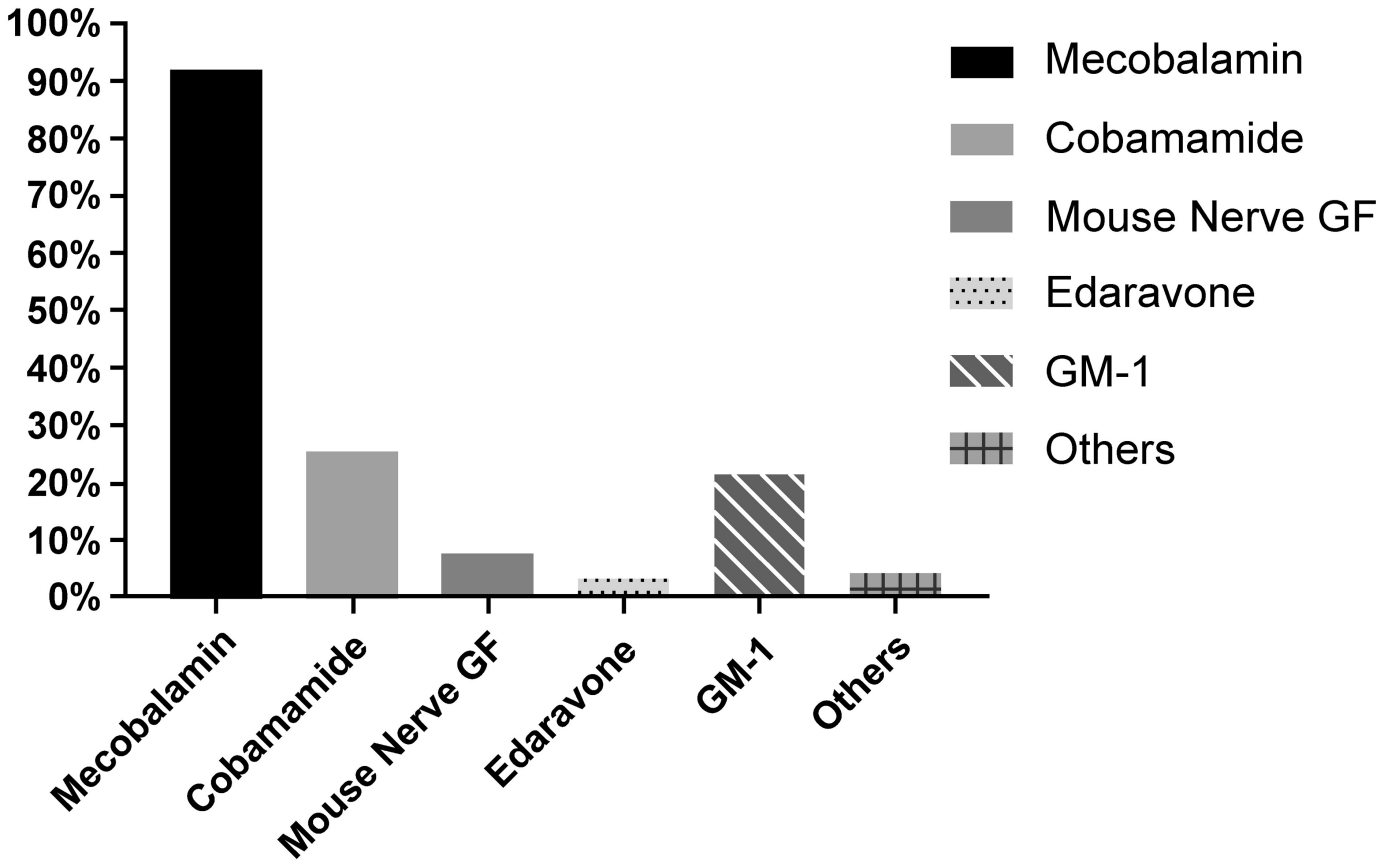
Application of neurotrophic drugs.

Neurogenic drugs were also considered a key treatment due to the risk of secondary neurological damage in SSNHL pathogenesis. Of the respondents, 80.7% (*n* = 1,626) included neurotrophic drugs as a combination treatment. The types of drugs are listed in Table 8. Almost all respondents indicated that mecobalamin should be included as a treatment for SSNHL (92.0%, *n* = 1,816). Approximately one-fourth of respondents suggested cobamamide (24.2%, *n* = 497) and GM-1 (21.6%, *n* = 426) as treatments. Less than one-tenth of respondents preferred mouse nerve growth factor and edaravone (Figure 8).

**Table 8:** Neurogenic drugs used by Chinese otolaryngologists.

**Drugs**	**Description**
Mecobalamin	A form of vitamin B12 used for peripheral neuropathy.
Cobamamide	An active form of vitamin B12 used for peripheral neuropathy.
Mouse nerve GF	Isolated from mouse submaxillary glands and used for regulating neuronal survival and development.
Edaravone	An antioxidant used as a free radical scavenger.
GM-1	Monosialotetrahexosylganglioside, a member of the ganglio series used for neuronal plasticity and repair.

## 4. Discussion

### 4.1. Analogical diagnostic criteria

The current criteria for SSNHL are not uniform internationally. Japan (4) and the European International Federation of Otorhinolaryngology Societies (IFOS) Conference (5) defined SSNHL as ≥ 30 dB hearing loss in at least three consecutive frequencies, whereas the clinical guidelines of the United States (6) and Spain (7) have expanded these criteria. In contrast, the clinical guidelines of Germany and the United Kingdom (8) do not specify a hearing loss threshold for SSNHL diagnosis. China initiated a multi-center randomized controlled trial (RCT) in 2008; the results were reported in 2015 (13). In the same year, a new edition of the guidelines (9) was published with reference to the latest international literature and the RCT results reported by Yu and Yang [13]. Based on these data, the diagnostic criteria were defined as hearing loss ≥ 20 dB in two consecutive frequencies, occurring within 72 h.

Our survey revealed that Chinese otologists exhibited strong heterogeneity in SSNHL diagnosis. When asked about the audiological criteria, one-third and one-fifth of the respondents diagnosed SSNHL based on the guidelines of China and the USA, respectively. However, when asked, “In which situation will you apply treatment to patients who suffer from sudden hearing loss,” more respondents (42.7%) selected the Chinese guidelines for audiological criteria, which is milder than American standards, showing a mismatch between diagnostic criteria and treatment criteria. Comparison with surveys in the UK exhibited a relatively consistent opinion regarding diagnosis. The majority (70%) of respondents defined SSNHL according to the criteria in the relevant guidelines. The reasons underlying the heterogeneity in diagnosis warrant further exploration, but the inconsistencies in audiological diagnostic criteria may reduce the quality of evidence of RCTs in China. When using milder criteria, the outcome of RCTs may become better than those who used the severer criteria, for the higher probability of self-recovery in mild cases.

### 4.2. Clinical classification

With a deeper understanding of SSNHL pathogenesis, clinical classification has become crucial. The German and Japanese guidelines classify SSNHL into five and four types, respectively. Similar to the Japanese guidelines, the Chinese guidelines classify SSNHL into four types based on clinical practice and recommend different treatment options and prognostic evaluations for each type. Although guidelines in the US, UK, and Spain do not propose specific classifications, possibly due to the lack of high-quality clinical evidence and cost of diagnostic tests, the IFOS Conference (5) has clearly emphasized the heterogeneity of SSNHL. Our survey revealed that most of the respondents supported the classification of the SSNHL, but the number of doctors who performed classification diagnoses in clinical practice was limited. Since clinical classification is predominantly based on PTA, which only requires basic medical equipment, the key factor affecting doctors' practices may not be the lack of devices caused by economic differences; rather, it may be due to insufficient understanding of the discipline in lower-level hospitals. We speculate that an appropriate classification could make the treatment more targeted and help to get more valuable results in clinical research because the prognosis of patients with different types could vary wildly.

### 4.3. Steroid therapies

Corticosteroids are a common treatment for SSNHL. A previous RCT (14) demonstrated the effectiveness of the systemic application of steroids for SSNHL. In contrast, Nosrati and Cinamon reported no significant difference in efficacy between steroid-treated and control groups (15), highlighting the need to verify the effectiveness of steroid treatment for SSNHL. In consideration of steroid efficacy and the consequences of permanent hearing loss, guidelines in most countries still recommend steroids as a treatment of choice. Nevertheless, the dose, timing, and frequency of treatment vary among countries. Chinese guidelines recommend the administration of prednisone at 1 mg/kg/d for 3 days, continuing for 2 additional days if effective, and tapering is not recommended. The heterogeneity of RCTs is a key factor resulting in the inconsistent systemic application of steroids.

Local administration of steroids in the inner ear remains a challenging field of research. Intratympanic steroids (ITS) have become the most commonly used local drug delivery system internationally (16). However, the strength of recommendations and treatment protocols vary among countries. ITS was commonly used as salvage treatment (17) but has recently been trialed as an initial treatment for SSNHL (18).

Compared with ITS, PAS is considered more economical, more convenient, minimally invasive, and has no inferiority compared with ITS (12). As first proposed and widely used by Chinese doctors, PAS constitutes a novel approach for local inner ear drug delivery. Clinical trials have reported satisfactory efficacy of PAS therapy, especially for intractable low-frequency sudden hearing loss, though the evidence remains insufficient due to defective trial design (19).

MRI assessments have demonstrated that the signal intensities of gadolinium-enhanced images of the cochlea were higher and longer following PAS administration compared to those following intravenous injection in guinea pigs (12). Recent studies have reported that PAS administration resulted in higher dexamethasone concentrations and longer durations in perilymph compared to systemic administration (20, 21). Notably, ITS administration and PAS resulted in greater fluorescence intensity in the basal portions of the organ of Corti and the scala media in the apical portions and stria vascularis, respectively. Theoretically, drugs injected post-auricularly may enter the inner ear through various pathways, such as circulation, tissue channels, and the sigmoid sinus (22–24). In contrast, for ITS, the drug enters the inner ear predominantly *via* the round window and oval window (25). Thus, different administration routes of local drug delivery may act on distinct targets in the inner ear, resulting in different clinical outcomes.

In China, both intratympanic and post-aural administration is recommended as a salvage option after systemic administration of corticosteroids. The use of steroid therapies by Chinese physicians was heterogeneous, especially in local applications. ITS was widely used as a salvage or initial treatment program. Despite not being recommended as an initial treatment in Chinese guideline (9), studies have reported its potential effectiveness (26, 27). In this survey, more Chinese physicians used post-auricular injection of steroids as a local drug delivery treatment instead of ITS. Clinical studies have demonstrated the effectiveness of post-auricular administration as a salvage treatment (28) and initial treatment (29) for SSNHL. Our results exhibited a similar or superior efficacy to systemic administration, especially for cases with low-frequency hearing loss. In this regard, post-auricular injections may replace or supplement ITS in China.

### 4.4. Combination therapy

The combination of other treatments with steroid therapy for SSNHL treatment remains controversial. A Cochrane systematic review reported inconclusive findings regarding hemorheology and vasodilators (30), predominantly due to inadequate RCTs. References have a strong heterogeneity with regard to drug types and efficacy assessments, making the interpretation of outcomes difficult. There is currently insufficient evidence to recommend drugs other than glucocorticoids for SSNHL treatment. Nevertheless, Chinese otolaryngologists still tended to select a combination treatment significantly different from the recommendations of US guidelines. In this survey, nearly all physicians indicated that cochlear ischemia and/or vasospasms were key factors in SSNHL pathogenesis. Ginkgo biloba extract and batroxobin were the most popular drugs applied by Chinese otolaryngologists. Most of the clinical studies supporting the efficacy of these drugs were performed by Chinese researchers, but the quality of studies included in the meta-analysis was low (31–33). Therefore, higher-quality RCTs assessing the effectiveness of hemorheological drugs are warranted.

### 4.5. Significance and limitations

Our survey was the first to investigate current opinions and clinical practices of SSNHL in China. We found that PAS therapy was widely used in China as a simple and practical choice, which costs significantly lower than ITS, and has better effects than ITS in the lower frequency of hearing threshold. Besides, otolaryngologists in China supported that the combination therapy of hemorheology drugs could improve the prognosis of SSNHL.

However, due to China's complex and wild regional distribution, the sample distribution in this study was slightly lower than expected in developed regions and higher than expected in moderately developed regions. These differences may slightly affect the accuracy of the results. In order to improve research quality, we conducted multifactorial analysis according to hospital level, physician level, and other aspects to reduce the impact of bias on the sample distribution.

## 5. Conclusions

This survey revealed Chinese otolaryngologists' views and clinical practices in the diagnosis and treatment of SSNHL. Chinese physicians exhibit substantial heterogeneity in SSNHL diagnostic criteria. Physicians generally support clinical classifications, but this requires improvements in actual clinical practice. In terms of steroid therapy, commonly used systemic administration strategies by Chinese physicians include short-term (5-day) therapy. For local administration, physicians generally employ ITS and PAS treatments as a salvage or initial protocol. The combination of hemorheology and neurotrophic drugs is widely used among Chinese physicians.

## Data availability statement

The original contributions presented in the study are included in the article/Supplementary material, further inquiries can be directed to the corresponding authors.

## Ethics statement

The studies involving human participants were reviewed and approved by the Ethics Committee of the Peking University People's Hospital. The patients/participants provided their written informed consent to participate in this study.

## Author contributions

NC, NK, and XinM wrote the main manuscript text. XN, XiaL, JS, ZJ, and XiuM prepared Figures 1–8. XiuL, SZ, QS, JL, and GC prepared Tables 1–6. MD and LY designed the questionnaire. All authors contributed to the article and approved the submitted version.

## References

[1] De Kleyn A. Sudden complete or partial loss of function of the octavus-system in apparently normal persons. Acta Otolaryngol. (1944) 32:407–29. 10.3109/00016484409119921

[2] Kuhn M Heman-Ackah SE Shaikh JA Roehm PC. Sudden sensorineural hearing loss: a review of diagnosis, treatment, and prognosis. Trends Amplif. (2011) 15:91–105. 10.1177/1084713811408349 21606048 PMC4040829

[3] Michel O. Deutsche gesellschaft für hals-nasen-ohren-heilkunde, kopf und hals chirurgie. Laryngorhinootologie. (2011) 90:290–3. 10.1055/s-0031-1273721 21560090

[4] Nakashima T Sato H Gyo K Hato N Yoshida T Shimono M. Idiopathic sudden sensorineural hearing loss in Japan. Acta Otolaryngol. (2014) 134:1158–63. 10.3109/00016489.2014.919406 25315915 PMC4266072

[5] Marx M Younes E Chandrasekhar SS Ito J Plontke S O'Leary S. International consensus (ICON) on treatment of sudden sensorineural hearing loss. Eur Ann Otorhinolaryngol Head Neck Dis. (2018) 135:S23–8. 10.1016/j.anorl.2017.12.011 29396226

[6] Chandrasekhar SS Tsai Do BS Schwartz SR Bontempo LJ Faucett EA Finestone SA. Clinical practice guideline: sudden hearing loss (update). Otolaryngol Head Neck Surg. (2019) 161:S1–45. 10.1177/0194599819859885 31369359

[7] Herrera M Berrocal JRG Arumí AG Lavilla MJ Plaza G. Update on consensus on diagnosis and treatment of idiopathic sudden sensorineural hearing loss. Acta Otorrinolaringol. (2019) 70:290–300. 10.1016/j.otoeng.2018.04.007 30093087

[8] Rauch SD. Clinical practice. Idiopathic sudden sensorineural hearing loss. N Engl J Med. (2008) 359:833–40. 10.1056/NEJMcp0802129 18716300

[9] Editorial Board of Chinese Journal of Otorhinolaryngology Head and Neck Surgery; Society of Otorhinolaryngology Head and Neck Surgery, Chinese Medical Association. Guideline of diagnosis and treatment of sudden deafness. Chin J Otorhinolaryngol Head Neck Surg. (2015) 50:443–447. 10.3760/cma.j.issn.1673-0860.2015.06.002 26695792

[10] Sutton L Schartinger V Url C Schmutzhard J Lechner D Kavasogullari C. Intratympanic steroid use for idiopathic sudden sensorineural hearing loss: current otolaryngology practice in Germany and Austria. Eur Arch Otorhinolaryngol. (2018) 275:1103–10. 10.1007/s00405-018-4958-8 29605865

[11] Lechner M Sutton L Ferguson M Abbas Y Sandhu J Shaida A. Intratympanic steroid use for sudden sensorineural hearing loss: current otolaryngology practice. Ann Otol Rhinol Laryngol. (2019) 128:490–502. 10.1177/0003489419828759 30770021

[12] Li J Yu L Xia R Gao F Luo W Jing Y. Postauricular hypodermic injection to treat inner ear disorders: experimental feasibility study using magnetic resonance imaging and pharmacokinetic comparison. J Laryngol Otol. (2013) 127:239–45. 10.1017/S0022215113000017 23406669

[13] Yu L Yang S. Multi-center clinical study promote the revision of sudden deafness guideline. Chin J Otorhinolaryngol Head Neck Surg. (2015) 50:441–2. 10.3760/cma.j.issn.1673-0860.2015.06.001 26695791

[14] Wilson WR Byl FM Laird N. The efficacy of steroids in the treatment of idiopathic sudden hearing loss. A double-blind clinical study. Arch Otolaryngol. (1980) 106:772–6. 10.1001/archotol.1980.00790360050013 7002129

[15] Nosrati-Zarenoe R Hultcrantz E. Corticosteroid treatment of idiopathic sudden sensorineural hearing loss: randomized triple-blind placebo-controlled trial. Otol Neurotol. (2012) 33:523–31. 10.1097/MAO.0b013e31824b78da 22429944

[16] El Kechai N Agnely F Mamelle E Nguyen Y Ferrary E Bochot A. Recent advances in local drug delivery to the inner ear. Int J Pharm. (2015) 494:83–101. 10.1016/j.ijpharm.2015.08.015 26260230

[17] Li H Feng G Wang H Feng Y. Intratympanic steroid therapy as a salvage treatment for sudden sensorineural hearing loss after failure of conventional therapy: a meta-analysis of randomized, controlled trials. Clin Ther. (2015) 37:178–87. 10.1016/j.clinthera.2014.11.009 25542075

[18] Filipo R Attanasio G Russo FY Viccaro M Mancini P Covelli E. Intratympanic steroid therapy in moderate sudden hearing loss: a randomized, triple-blind, placebo-controlled trial. Laryngoscope. (2013) 123:774–8. 10.1002/lary.23678 23378346

[19] Jing Y Yu L Ma X. Efficacy of postauricular methylprednisolone administration for refractory sudden hearing loss. Chin J Otol. (2014) 12:452–4. 10.3969/j.issn.1672-2922.2014.03.026

[20] Lin YJ Yu LS. The concentration analysis of dexamethasone in rats' inner ear tissue after postaurieal injection and muscle injection. Chin J Otolaryngol Head Neck Surg. (2009) 16:381–4.

[21] Wang Y Han L Diao T Jing Y Wang L Zheng H. comparison of systemic and local dexamethasone administration: from perilymph/cochlea concentration to cochlear distribution. Hear Res. (2018) 370:1–10. 10.1016/j.heares.2018.09.002 30223171

[22] Salt AN Hirose K. Communication pathways to and from the inner ear and their contributions to drug delivery. Hear Res. (2018) 362:25–37. 10.1016/j.heares.2017.12.010 29277248 PMC5911243

[23] Yamasoba T Yagi M Roessler BJ Miller JM Raphael Y. Inner ear transgene expression after adenoviral vector inoculation in the endolymphatic sac. Hum Gene Ther. (1999) 10:769–74. 10.1089/10430349950018526 10210144

[24] Colletti V Mandalà M Carner M Barillari M Cerini R Pozzi Mucelli R. Evidence of gadolinium distribution from the endolymphatic sac to the endolymphatic compartments of the human inner ear. Audiol Neurootol. (2010) 15:353–63. 10.1159/000292929 20215744

[25] Zou J Ramadan UA. Pyykkö I. Gadolinium uptake in the rat inner ear perilymph evaluated with 47 T MRI: a comparison between transtympanic injection and gelatin sponge-based diffusion through the round window membrane. Otol Neurotol. (2010) 31:637–41. 10.1097/MAO.0b013e3181d2f095 20142794

[26] Rauch SD Halpin CF Antonelli PJ Babu S Carey JP Gantz BJ. Oral vs intratympanic corticosteroid therapy for idiopathic sudden sensorineural hearing loss: a randomized trial: a randomized trial. JAMA. (2011) 305:2071–9. 10.1001/jama.2011.679 21610239

[27] Hobson CE Alexander TH Harris JP. Primary treatment of idiopathic sudden sensorineural hearing loss with intratympanic dexamethasone. Curr Opin Otolaryngol Head Neck Surg. (2016) 24:407–12. 10.1097/MOO.0000000000000288 27379547

[28] Yang XQ Yu LS Ma X. Postaurical injection of compound betamethasone to treat the intractable low-frequency sensorineural hearing loss. Chin J Otolaryngol Head Neck Surg. (2007) 42:814–6. 10.3760/j.issn:1673-0860.2007.11.004 18300441

[29] Wang M Fan Z Hou Z Zhang D Wang H. Topical injection and systemic application of glucocorticoids in the treatment of idiopathic sudden sensorineural hearing loss by type. Chin J Otolaryngol Head Neck Surg. (2014) 49:11–5. 10.3760/cma.j.issn.1673-0860.2014.01.004 24680330

[30] Wei BPC Stathopoulos D O'Leary S. Steroids for idiopathic sudden sensorineural hearing loss. Cochrane Database Syst Rev. (2013) CD003998:1–37. 10.1002/14651858.CD003998.pub3 23818120 PMC7390468

[31] Li Y. Interventions in the management of blood viscosity for idiopathic sudden sensorineural hearing loss: a meta-analysis. J Health Res Rev. (2017) 4:50. 10.4103/jhrr.jhrr_125_16

[32] Gong Y Liang C Li J Tian A Chen N. Vasodilators for sudden sensorineural hearing loss: a systematic review of randomized controlled trials. Chin J Otolaryngol Head Neck Surg. (2002) 37:64–8. 12768798

[33] Feng T Zhang Q Wei J Wang X Geng Y. Effects of alprostadil combined with hyperbaric oxygen on hearing recovery and hemorheology in patients with sudden sensorineural hearing loss and analysis of related influencing factors. Exp Ther Med. (2022) 23:242. 10.3892/etm.2022.11167 35222719 PMC8815044

