# Supplementary material for: Diagnosis, differential diagnosis, and treatment for sudden sensorineural hearing loss: Current otolaryngology practices in China
Source: Front Neurol. 2023 Feb 23;14:1121324. doi: 10.3389/fneur.2023.1121324 (PMC9995834; doi:10.3389/fneur.2023.1121324)

**Supplementary Material 1: Questionnaire**

***The questionnaire was modified from the article: *Intratympanic Steroid Use for Sudden Sensorineural Hearing Loss: Current Otolaryngology Practice (Lechner, 2019)* and *Intratympanic steroid use for idiopathic sudden sensorineural hearing***

***loss: current otolaryngology practice in Germany and Austria (Sutton, 2018)***

1. The hospital you are working at is classified as [single select question]

A. Provincial hospital

B. City hospital

C. County hospital

D. Others

2. What is the grade of your hospital? [single select question]

A. 3-A

B. 3-B

C. 2-A

D. 2-B

E. Others

3. How long have you worked in otorhinolaryngology? [single select question]

A. Less than 5 years

B. 5-10 years

C. 10-20 years

D. 20-35 years

E. More than 35 years

4. What is your current professional title? [single select question]

A. Student

B. Resident

C. Specialist

D. Senior consultant

E. Consultant

5. What is your current specialty? [single select question]

A. Otology

B. Rhinology

C. Laryngology, head and neck

D. Unspecified, mainly otology

E. Undetermined

F. Others

6. What is your main focus in otology? [single select question]

A. Otologic surgery

B. Internal medicine of otology

C. Basic research

D. Undetermined

E. Others

7. On average, how often do you treat sudden sensorineural hearing loss (SSNHL) patients? [single select question]

A. More than 10 cases per week

B. 5-10 cases per week

C. About 2-3 cases per week

D. About 2-3 cases per month

E. Less than 1 case per month

F. Others

8. What is the most common type of SSNHL patients you encountered? [single select question]

A. Patients on their first visit who developed SSNHL for less than 14 days

B. Patients on their first visit who developed SSNHL for more than 14 days

C. Patients on their first visit who developed SSNHL for less than 7 days

D. Patients responding poorly to the initial treatment

E. Patients with different levels of hearing loss and various visit time

9. What routine examinations do you usually prescribe before the SSNHL diagnosis? [multi-select question]

A. No examinations. I make a diagnosis only based on a patient’s chief complaints.

B. Otoscope

C. Pure tone audiometry

D. Impedance audiometry

E. ABR

F. Imaging

G. Vestibular function examination

H. Electrocochleogram

10. In your opinion, what is the best criterion for SSNHL diagnosis? [single select question]

A. A 30 dB loss in at least 3 frequencies

B. A 20 dB loss in at least 3 frequencies

C. A 20 dB loss in any 2 frequencies

D. A 30 dB loss in any frequency

E. A 20 dB loss in any frequency

F. Other

11. In practice, what indication do you use for infusion therapy for SSNHL? [multi-select question]

A. A 30 dB loss in at least 3 frequencies

B. A 20 dB loss in at least 3 frequencies

C. A 20 dB loss in any 2 frequencies

D. A 20 dB loss or more in any frequency

E. Patients’ subjective complaints of hearing loss without objective hearing loss in audiometry

F. Others

12. In practice, what indication do you use for infusion therapy for SSNHL? [multi-select question]

A. A 30 dB loss in at least 3 frequencies

B. A 20 dB loss in at least 3 frequencies

C. A 20 dB loss in any 2 frequencies

D. A 20 dB loss or more in any frequency

E. Patients’ subjective complaints of hearing loss without objective hearing loss in audiometry

F. Others

13. In your opinion, what is the effect of classifying SSNHL on subsequent treatment? [multi-select question]

A. I can explain to the patients their conditions and possible causes based on the classification.

B. I can predict the prognosis of SSNHL based on the classification.

C. I can choose different treatment options based on the classification.

D. There is no effect or I do not make classifications.

14. What pathogenesis of SSNHL do you accept? [multi-select question]

A. Viral infection

B. Blood supply disorder or vasospasm

C. Endolymphatic hydrops

D. Nerve injury

E. Immune response or inflammation

F. Migraine mechanism

15. What are the most common route and dose when you administrate systemic steroids? [single select question]

A. Intravenous administration for 5 days

B. Oral administration for 5 days

C. Intravenous administration and then tapering off

D. Intravenous administration with subsequent oral administration and then tapering off

E. Oral administration and then tapering off

16. What drugs do you usually choose when administrating steroids intravenously? [multi-select question]

A. Methylprednisolone

B. Dexamethasone

C. Hydrocortisone

D. Others

17. What drugs do you usually choose when administrating steroids orally? [multi-select question]

A. Methylprednisolone

B. Dexamethasone

C. Hydrocortisone

D. Others

18. Have you ever used intratympanic steroids to treat SSNHL? [single select question]

A. Yes

B. No

19. Under what circumstances will you use intratympanic steroid therapy? [multi-select question]

A. As first-line treatment for all patients.

B. As first-line treatment for selected patients.

C. For salvage for all patients after failed systemic steroid therapy.

D. For salvage for selected patients after failed systemic steroid therapy.

20. Do you treat all the SSNHL patients with hemorheologic therapy? [single select question]

A. Yes

B. No

21. What are your preferred drugs? [multi-select question]

A. Ginkgo biloba extract

B. Alprostadil

C. Batroxobin

D. Vinpocetine

E. Shuxuetong

F. Xueshuangtong

G. Others

22. In your opinion, what drugs have the best efficacy in clinical practice? [multi-select question]

A. Ginkgo biloba extract

B. Alprostadil

C. Batroxobin

D. Vinpocetine

E. Shuxuetong

F. Xueshuangtong

G. Others

23. Do you treat all the SSNHL patients with nerve nourishing therapy? [single select question]

A. I apply it to almost all the patients.

B. I apply it to selected patients.

C. I seldom apply it.

24. What are your preferred drugs? [multi-select question]

A. Mecobalamin

B. Cobamamide

C. Suetaisheng (Mouse Nerve Growth Factor)

D. Edaravone

E. Monosialotetrahexosylganglioside

F. Others

**Supplementary Material 2**

The administrative division map of China and the schematic diagram of sample size in each province.


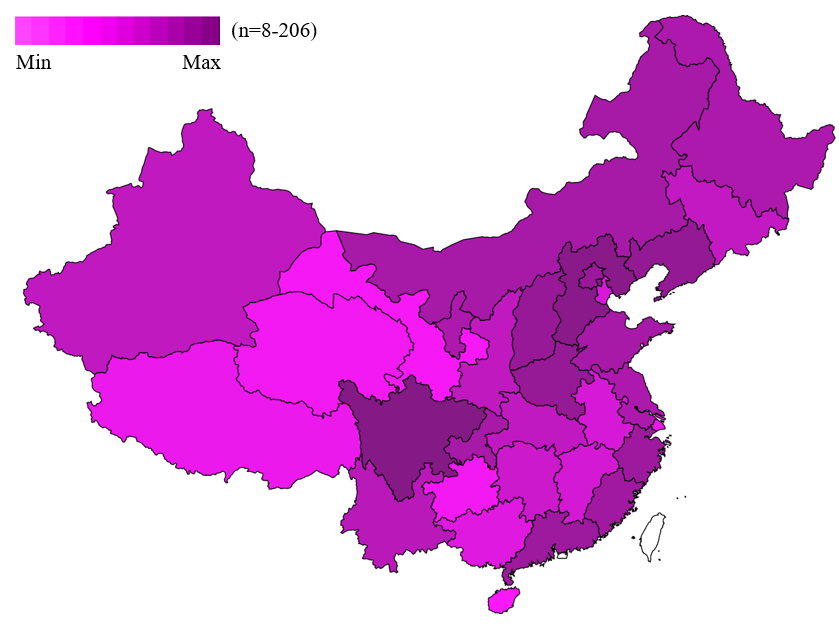

Supplement: Supplementary file 1 [file Data_Sheet_1.docx]
